# The effects of t-bomb II on lean body mass and hormonal profile in resistance trained athletes

**DOI:** 10.1186/1550-2783-11-S1-P44

**Published:** 2014-12-01

**Authors:** Matthew H Sharp, Ryan P Lowery, Jeremy E Silva, Jacob T Rauch, Sean A McCleary, Jacob A Ormes, Kevin A Shields, John I Georges, Jacob M Wilson

**Affiliations:** 1 The University of Tampa, Tampa, Florida, USA

## Background

Periodization describes an organized approach to training variation throughout a given year. For competitive athletes this typically includes and offseason, pre-season and in-season. The offseason is meant to build muscle, increase power, strength and endurance. However, the season brings on stress that can negatively impact body composition via decreasing lean body mass (LBM). One general cause may be a decline in anabolic hormones, particularly testosterone. T Bomb II is a product made by Maximum Human Performance, INC. (MHP). It is a proprietary blend of ingredients such as fenugreek extract which has been shown to elevate testosterone levels. PURPOSE: Therefore, the purpose of this study was to investigate the 6 week impact of T Bomb II (TB) supplementation on the ability to maintain testosterone and LBM during season.

## Methods

20 resistance trained NCAA National Championship baseball athletes (age 22.1 ± 1.9 years, mass 69.89 ± 6.6 kg, height 180.8 ± 13.1 cm) volunteered to participate in this study and were given supplementation of either TB (n=10) or placebo (n=10) for six weeks taking 3 capsules twice a day. All subjects participated in supervised resistance training three days a week and all subjects were on controlled diet (50% CHO, 25% FAT, 25% PRO). Duel X-Ray Absorptiometry (DXA) scans were performed at 0 and 6 weeks to determine LBM. Hormonal and safety panels including free testosterone (FT), total testosterone (TT), IGF-1 and total estrogen (TEE) were collected at weeks 0 and 6. Consent to publish the results was obtained from all participants.

## Results

The ability to sustain LBM was significant in the TB group (-0.2 kg) compared to the placebo group (-1.5 kg). The FT collection resembled a 16.3% increase in the TB group (13.28 ± 3.32 to 15.45 ± 3.22 ng/dL). The TT increased significantly in the TB group (541.5 ± 153.89 to 639.1 ± 100.18) and decreased in the placebo group (554.5 ± 136.86 to 497.2 ± 110.91 ng/dL) a 18.02% and -8.25% change, respectively. The changes in IGF-1 and E at weeks 0 and 6 were insignificant for both groups.

## Conclusion

This study suggest that taking 3 capsules (one serving) of T-Bomb II twice daily can reduce the rate of LBM lost and increase FT and TT of competitive athletes during season.

